# Chinese Ideas Respecting the Anatomy of the Circulating System

**Published:** 1847-03

**Authors:** 


*Chinese ideas respecting the Anatomy of the Circulating System.*— The heart is figured low in the thorax, is considered a single cavity, and the reservoir of good things ; it has little active connection, none that can be traced with the general circulation. From above, the windpipe passes directly into it ; while from below, the tube of a second, elective stomach, connected with the first, recipient stomach, by some kind of conduit, enters it at nearly the same point with the windpipe. Short work is thus made with the complicated and mysterious process of assimilation. One vessel proceeds from the heart to the liver, and another descending along the course of the spine, after communication by a broad reservoir-like expansion, with the kidneys, *it is presumed*, terminates in the genitals. There is, besides, a double canal, connecting the heart at a point near its apex, with the tube last noticed. But what all those things mean, and what part, if any, the heart acts in propelling the blood, the professor who was asked to explain what appeared in the plate, failed to show. Chinese notions regarded the circulating system, as on most other subjects, are peculiar to themselves, and differ entirely from those entertained by European physiologists, anterior to the time of Harvey and Servetus. In this, as in other vital actions, they introduce the sexual system. They have some idea of difference between arteries and veins, but what it is, and what offices they assign to each, could not be ascertained.

The arteries are said to be male and female. Of the former, three, belonging to the hand, proceed from it to the head ; while three, belonging to the foot, originate in the hand, whence they descend to their proper place. On the other hand, three female arteries proceed from the bowels to the hand, to which they belong ; and three, belonging to the foot, originate there, and then proceed to the intestines.

Besides these vessels, there is the pulse artery, which moves with every respiration three inches, and performs its circuit in two minutes. The chief concern of the doctor is the study of this artery, that he may clearly understand, and accurately unfold its manifold meanings; for in so much as he has skill to read its language accurately, by so much is he excellent as a practitioner. From it, besides the temperament of the patient, he deduces the diagnostic character of the disease he is called to treat, after which, having settled the kind and degree of disorder which has arisen among the elements, he sets manfully to work, and boldly promises a cure, knowing, what is not peculiar to China, that the man who promises most in medicine is most followed.

The veins are said to circulate day and night, but it does not appear that they believe the motion to be onward and in a circle: for they say, that having arrived at the end, it again commences where it left off. Nor do they admit that the arteries and veins have any connection; for they allege that all the arteries and veins have their respective cavities, twelve in all, such as the liver and heart, from which they proceed, and corresponding places where they end; and that, throughout the body, there are paths, ducts, and channels, distinct and appropriate to each, so that the blood does not meet with obstruction in its passage, though why it passes, and by what mechanism, does not appear.

The heart is said to be the ruler, from which the spirits proceed; it is also held to be the receptacle of marrow, which comes from the brain, and goes to the reproductive organs. The lungs are vehicles by which the temper is regulated. The liver holds the place of a general, whence proceeds contrivances and orders. The bile acts as umpire, settling disputed points. The spleen performs the part of messenger, and is the fountain of joy—a function not before ascribed to it, but to which it has as good a title as to be considered the seat of ill-humour and despondency. The stomach is the granery of the body, and the governor of the five tastes. The great duct is the promoter of principles, and the operator of changes; the smaller ones being the receptacles of superfluity, where digestion is carried on. The kidneys are the rulers of strength, whence all skill proceeds. The bladder, having no connection with the kidneys, is the general reservoir of the absorbents.—*Wilson's Med. Notes on China.*

